# Exploring financial difficulty and help-seeking behaviour among medics in the United Kingdom: a cross-sectional survey

**DOI:** 10.1186/s12960-025-01008-0

**Published:** 2025-08-28

**Authors:** Monisha Edirisooriya, Milou Silkens, Asta Medisauskaite

**Affiliations:** 1 Research Department of Medical Education, University College London, Gower Street, London, England WC1E 6BT; 2 Erasmus School of Health Policy & Management (Room J6-35), Erasmus University, Burgemeester Oudlaan 50, 3062 PA Rotterdam, The Netherlands

**Keywords:** Medical students, Doctors, Financial difficulty, Help-seeking behaviour, Demographic characteristics

## Abstract

**Background:**

While an intensifying workforce crisis and industrial action across the United Kingdom (UK) healthcare system has shed light on financial strains medics in the UK may face, there remains a lack of evidence on how various groups among an increasingly diversifying profession may be affected. This study explored experiences of financial difficulties and help-seeking behaviours across different demographic groups of medics.

**Methods:**

The demographic characteristics, financial worries and difficulties, and help-seeking behaviours of 442 medical students and doctors in the UK were surveyed. Inferential statistics and regression analyses were undertaken in SPSS. Qualitative responses regarding improving help-seeking underwent content analysis.

**Results:**

Over 80% of participants reported ever worrying about their finances. One-third had ever experienced financial difficulty. Of these, there were a higher percentage of medics with a disability (53.4%) than without a disability (30.4%); and with caring roles (47.2%) compared to those without (30.4%). LGBTQ + participants were 3.5 times more likely to have ever worried about their financial situation compared to those identifying as heterosexual. Those with a non-UK Primary Medical Qualification (PMQ) were twice as likely to experience financial difficulty compared to UK-PMQ respondents. Education and workplace sources of financial help were more likely to be sought by those without a disability, those with a UK-PMQ, participants in the ≤ 25 age bracket and students in comparison to doctors. Participants with a non-UK PMQ, participants aged 36–45 years, and doctors were more likely to seek external support. The most common responses to improving early help-seeking stemmed around improving understanding of the available support, and reducing stigma.

**Conclusions:**

Experiences of financial insecurity among medics are extremely common. Our study has highlighted that LGBTQ + medics and those with a non-UK PMQ may be particularly vulnerable to financial problems as well as those with a disability or caring role. Education and workplace mechanisms of financial support may be underutilised by medics with disabilities, those with a non-UK PMQ, and those in postgraduate settings more broadly. Institutions should seek to improve awareness and accessibility of financial support.

**Supplementary Information:**

The online version contains supplementary material available at 10.1186/s12960-025-01008-0.

## Background

Medics in the United Kingdom (UK) are experiencing a different financial reality to their predecessors and are not absolved from facing financial challenges. Medical careers are fraught with costs at both the undergraduate and postgraduate level, a frequently attested reason for experiencing financial difficulty [1, 2]. A recent study found that 60.0% of surveyed medical students have experienced monetary pressures during their degree, with 65.0% of those affected experiencing a negative impact on their mental health as a result. Additionally, nearly half of all respondents reported either they or someone they knew had considered withdrawing from medicine as a direct result of financial pressure (47.0%) [3]. To combat these pressures, another study found 61.8% of medical students have cut down on spending on essential items, due to an insufficient student finance system, with an inadequate National Health Service (NHS) bursary particularly causing financial hardship for those from lower income backgrounds [4]. As newly qualified doctors enter Foundation training^1^ many start repaying significant university debts,^2^ alongside mandatory costs such as for indemnity, union membership, regulation (General Medical Council) and relocation. Additionally, doctors spend thousands of pounds undertaking courses, conferences, obtaining further qualifications and examinations as they prepare to apply to specialty training [5]. These obligatory costs continue to amass throughout their careers.

The current ‘cost of living crisis’ and pay erosion in the UK are two major factors currently exacerbating the risk of financial difficulty across the population. Doctors across all levels have incurred a significant real-terms pay cut by more than a quarter since 2008 [6]. A recent survey undertaken by the British Medical Association revealed 71.0% of nearly 4000 doctors-in-training reporting feeling ‘very worried’ about the impact of the cost of living [7]. Financial difficulty was common with doctors struggling to afford utilities (51.0%), essential travel (45.0%) and rent or mortgage costs (45.0%). Half of respondents had borrowed money from family or friends in the past year. Many healthcare professionals have since taken industrial action over fair pay across 2022–2023 [8]. Financial hardship can have significant personal, professional and wider workforce consequences. These include barriers to academic progression, failing examinations, and differential attainment (the variation in achievement between different groups of medics); stress, depression and burnout; and leaving the profession altogether [9–13]. The NHS is currently facing the ‘greatest workforce crisis in history’, with increasing service demand and long-term staff shortages [14]. Four in 10 doctors plan to leave the NHS as soon as they can secure a post elsewhere, with poor pay and undesirable working conditions reported as the key driving factors [15].

Given the conditions depicted above, ensuring those at risk of, or currently experiencing financial difficulty have early access to preventative and alleviating support is paramount. To achieve this, a demographic analysis of the affected population and a better understanding of their help-seeking behaviours is an important evidence gap to address. Socio-demographic factors are known to affect financial well-being [16]. Among the general population, factors precipitating financial hardship can include illness, disability, caring responsibilities and relationship breakdown [17] and this likely to be reflected within medics [18, 19]. Students from widening participation and low-income backgrounds may also be in financially vulnerable positions [20]. LGBTQ + students and doctors might also be vulnerable if they lose financial support from family, which research indicates is a common reason people fear coming out about their sexual orientation [21, 22]. Furthermore, to the author’s knowledge there are no studies exploring help-seeking behaviour for financial hardship within medics. Help-seeking behaviour can be characterised by problem focused, intentional action and interpersonal interaction [23]. Help-seeking behaviours are important to understand when implementing systems of support. It may be hypothesised that there is likely to be a difference in help-seeking behaviour depending on demographic characteristics, based on existing studies which largely focus on mental health [24].

Additionally, as financial hurdles have been outlined as a barricading factor to workforce diversity in medicine, affecting recruitment through to retention [25, 26], addressing this knowledge gap is a fundamental component in the strive of the NHS to become an exemplar of equality, diversity and inclusion. This is not only an important moral and legal responsibility to staff, but a diverse workforce representative of its community has been shown to benefit patient outcomes and system efficiency [27]. Resultantly, this study aims to explore the experiences of financial difficulties and help-seeking behaviours across different demographic groups of medics.

## Methods

### Ethics and study setting

Following receival of ethics permission from the University College London Research Ethics Committee (ref. 13,311/003), this cross-sectional questionnaire-based study was conducted between October 2021 and January 2022. The questionnaire excerpt used by the current study formed part of a larger survey study [28] commissioned by the Royal Medical Benevolent Fund (RMBF), which supports medical students and doctors experiencing financial difficulty secondary to age, illness, injury, disability, or bereavement. The study sought to investigate who is experiencing financial hardship within the profession, and secondly evaluate to what extent the RMBF meets the needs of this cohort. Medical students and doctors training and working within the UK were invited to complete an online survey on the RedCap platform. To assist with survey uptake, the following organisations were approached: medical schools; deaneries; widening participation schemes and national professional organisations. In addition, social media was used to increase survey participation. Employing this multi-strategy approach meant an accurate response rate could not be calculated, but in total 442 participants completed the survey out of 597 that started the survey (74%). Participants signed an informed consent sheet prior to completing the questionnaire. All responses were anonymised.

### Questionnaire design

The full questionnaire was devised using a range of open and closed questions and all questions were compiled by the authors of the original study incorporating feedback from the project advisory group, which included medical students, doctors, researchers, members from the RMBF and other supporting organisations. For the current study, subsets of the questionnaire were used for the analysis.

### Data collection

Data were collected on demographic characteristics including gender, sexuality, relationship status, ethnicity, disability, caring responsibilities, primary medical qualification (PMQ), age and career stage.

Regarding experiences of financial problems, we asked if participants (i) ever worried about their financial situation; (ii) ever experienced financial difficulties (inability to meet financial obligations) (both scored yes/no/prefer not to say).

Next, intentions to seek financial help were measured by presenting participants with ten sources of support grouped as follows: personal (partner, family, friends; Cronbach’s alpha = 0.529); education and work (professional organisations, workplace, student loan; Cronbach’s alpha = 0.593); external (bank, charity, loan, government; Cronbach’s alpha = 0.469). Despite lower than desired Cronbach’s alphas, all authors agreed to include these groupings in addition to the analysis of intentions to seek help from all sources combined. The authors hypothesised that the groupings could provide more in-depth information to the interested reader, and the use of alternative clusters were not meaningful. However, considering the lower reliability of these groupings the results based on these groupings should be interpreted with caution. Cronbach’s alpha of the overall support seeking scale is 0.602. We asked participants how likely they were to seek help from these resources if/when experiencing financial difficulties. The items were scored on a Likert scale ranging from 1 (extremely unlikely) to 7 (extremely likely), developed based on a validated questionnaire surveying help-seeking behaviour [29].

Finally, responses to two open ended questions exploring barriers to receiving support were collected to assist with future recommendations to improve support for medics in financial need. We asked: (i) What would have helped you to seek support or seek support earlier? (ii) If you did not seek help, could you tell us why not?

### Statistical analysis

All quantitative data were analysed using IBM Statistical Package of Social Sciences (SPSS). A *p*-value of < 0.05 was considered statistically significant for all analyses. Some demographic sub-groups were combined for analyses as described in Table 1, due to more detailed categories being populated with limited data points.

**Table 1:** Demographic characteristics of responders

Demographic group	Sub-group	Participant frequencies	Sub-groups used in analysis
(N = 442)		N (%)	(* and **= combined sub-groups for analysis)
Gender	Male	134 (30.3)	Male
	Female	305 (69.0)	Female
	Prefer not to say	3 (.7)	
Sexuality	Bisexual*	34 (7.7)	LGBTQ + *
	Gay/lesbian*	20 (4.5)	Heterosexual
	Heterosexual	363 (82.1)	
	Other*	3 (.7)	
	Prefer not to say	21 (4.8)	
	Missing	1 (.2)	
Relationship status	Single and never married*	209 (47.3)	Single*
	Divorced*	14 (3.2)	Relationship**
	Married/civil partner**	123 (27.8)	
	Cohabiting**	78 (17.6)	
	Prefer not to say	5 (1.1)	
	Other (separated)*, (relationship)**	12 (2.7)	
	Missing	1 (.2)	
Ethnicity	Arab or Arab British*	9 (2.1)	Ethnic minority*
	Asian or Asian British*	72 (16.4)	White
	Black African, Caribbean, or Black British*	14 (3.2)	
	Mixed or multiple ethnic groups*	25 (5.7)	
	Other*	4 (.9)	
	Prefer not to say	7 (1.6)	
	White	307 (69.5)	
	Missing	4 (.9)	
Disability status	Disability	58 (13.1)	Disability
	No disability	376 (85.1)	No disability
	Prefer not to say	7 (1.6)	
	Missing	1 (.2)	
Carer status	Carer	91 (20.6)	Carer
	Not a carer	342 (77.4)	Not a carer
	Prefer not to say	5 (1.1)	
	Missing	4 (.9)	
PMQ	UK	388 (87.8)	UK
	Non-UK	51 (11.5)	Non-UK
	Missing	3 (.7)	
Career stage	Medical student	200 (45.2)	Medical student
	Foundation year	15 (3.4)	Foundation year
	Specialty training	102 (23.1)	Specialty training
	GP	31 (7.0)	GP
	Consultant	54 (12.2)	Consultant
	Other doctor	39 (8.8)	Other doctor
	Missing	1 (.2)	
Age groups	≤ 25	111 (25.1)	≤ 25
	26–35	113 (25.6)	26–35
	36–45	37 (8.4)	36–45
	46–55	31 (7.0)	46–55
	56 +	12 (2.7)	56 +
	Missing	138 (31.2)	

First, data exploration was undertaken using inferential statistics. An exploratory demographic analysis of participants ever having worried about/ experienced financial difficulty, was completed using Pearson Chi-square tests (Appendix A). Pearson Chi-square tests, T-tests and one-way analysis of variance (ANOVA) were used to compare seeking support across different demographic groups (Appendices B–F; scales were normally distributed: skewness and kurtosis −1 to 1, with no extreme outliers).

For the main study analyses, binary logistic regression was used to test if and how demographic characteristics link with financial worries and experiencing financial difficulty providing odd ratios (Table 2). These were the only two survey questions asked in past tense (‘Ever worried about financial situation?’ and ‘Ever experienced financial difficulties?), therefore we limited the regression analyses to include four demographic characteristics: gender, sexuality, ethnicity and PMQ. This was due to the inaccuracy that may come with inputting data for the remaining five demographic characteristics which (are more likely to) change over time: relationship status, disability status, carer status, age, and career stage.

**Table 2:** Predicting worrying about and experiencing financial problems based on demographic characteristics using binary logistic regression

Group (reference group)	Coefficient	Standard error	Wald	df	p	Odds ratio	95% CI for OR Lower	95% CI for OR Upper
*Ever worried about financial situation* (N = 408)								
Gender (Male)	− .060	.299	.040	1	.841	.942	.524	1.692
*Sexuality (Heterosexual)*	**1.265**	**.612**	**4.272**	**1**	.039*	**3.542**	**1.068**	**11.754**
Ethnicity (White)	− .497	.295	2.839	1	.092	.608	.341	1.084
PMQ (UK PMQ)	− .216	.402	.289	1	.591	.805	.366	1.772
*Ever experienced financial difficulty* (N = 401)								
Gender (Male)	− .114	.231	.245	1	.621	.892	.567	1.403
Sexuality (Heterosexual)	.003	.312	.000	1	.992	1.003	.544	1.849
Ethnicity (White)	− .361	.252	2.050	1	.152	.697	.425	1.142
*PMQ (UK PMQ)*	**.692**	**.335**	**4.268**	**1**	.039*	**1.998**	**1.036**	**3.853**

^*^ statistically significant (*p *< .05)

Bold rows indicate significant results

Linear regression analysis was then completed to test the relationship between demographic characteristics and the types of help that have been or would be sought to alleviate financial difficulty (Table 3). Multicollinearity was between 1 to 1.415 variance inflation factor for all demographic variables for all analyses, therefore all variables were retained. An a priori power analysis was conducted using G*Power version 3.1 to determine the minimum sample size required to achieve 90% power for detecting a medium effect size (0.15) at a significance criterion of *a* = 0.05 was *N* = 141 for linear multiple regression. Thus, the study sample size was sufficient.

**Table 3:** Linear regression analysis showing the relationship between demographic groups and sources of help likely to be sought

Demographic group	Comparative	Variables in model	Coefficient	Standard error	Standardised coefficients: Beta	t	p
*All sources of help*							
Gender	Male	Female	.122	.082	.072	1.476	.141
Sexuality	Heterosexual	LGBTQ +	− .220	.114	− .094	−1.933	.054
Relationship status	Relationship	Single	− .045	.093	− .029	− .489	.625
Ethnicity	White	Ethnic minority	.022	.086	.013	.259	.795
Disability status	No disability	Disability	− .176	.114	− .075	1.547	.123
PMQ	UK	Non-UK	− .003	.121	− .001	− .022	.982
Carer status	Non-carer	Carer	− .062	.103	− .032	− .599	.550
Age group	≤ 25	26–35 36–45 46–55 56 +	.101 .075 − .200 .269	.098 .151 .163 .249	.056 .027 − .065 .054	1.035 .496 − 1.230 1.082	.301 .620 .220 .280
Career stage	Students	Doctors	− .108	.102	− .068	− 1.055	.292
*Personal sources of help (partner, friends, family)*							
Gender	Male	Female	.060	.123	.024	.490	.624
Sexuality	Heterosexual	LGBTQ +	− .233	.170	− .067	− 1.372	.171
Relationship status	Relationship	Single	− .167	.139	− .071	− 1.200	.231
Ethnicity	White	Ethnic minority	.174	.129	.067	1.350	.178
Disability status	No disability	Disability	− .159	.170	− .046	− .937	.349
PMQ	UK	Non-UK	.050	.180	.014	.278	.781
Carer status	Non-carer	Carer	− .119	.154	− .041	− .773	.440
Age group	≤ 25	26–35 36–45 46–55 56 +	.073 − .175 − .359 − .148	.146 .226 .243 .372	.027 − .041 − .079 − .020	.502 − .773 −1.474 − .398	.616 .440 .141 .691
Career stage	Students	Doctors	.210	.153	.090	1.377	.169
*External sources of help (bank, charity, loan, government)*							
Gender	Male	Female	.095	.105	.043	.907	.365
Sexuality	Heterosexual	LGBTQ +	− .227	.145	− .075	−1.562	.119
Relationship status	Relationship	Single	.002	.119	.001	.016	.987
Ethnicity	White	Ethnic minority	− .090	.110	− .039	− .815	.416
Disability status	No disability	Disability	− .058	.145	− .019	− .398	.691
**PMQ**	**UK**	**Non-UK**	**.315**	**.154**	**.099**	**2.047**	.041*
Carer status	Non-carer	Carer	− .121	.131	− .048	− .917	.359
**Age group**	** ≤ 25**	26–35 **36–45** 46–55 56 +	.100 **.465** .203 .448	.125 **.193** .208 .317	.043 **.126** .051 .069	.801 **2.415** .976 1.412	.424 .016* .330 .159
**Career stage**	**Students**	**Doctors**	**.350**	**.131**	**.170**	**2.680**	.008*
*Education and work sources of help (professional organisations, workplace, student loan)*							
Gender	Male	Female	.218	.129	.075	1.691	.092
Sexuality	Heterosexual	LGBTQ +	− .207	.178	− .052	− 1.163	.246
Relationship status	Relationship	Single	.017	.146	.006	.114	.909
Ethnicity	White	Ethnic minority	.021	.135	.007	.153	.879
**Disability status**	**No disability**	**Disability**	** − .372**	**.178**	** − .093**	− **2.095**	.037*
**PMQ**	**UK**	**Non-UK**	** − .454**	**.189**	** − .108**	− **2.405**	.017*
Carer status	Non-carer	Carer	.072	.161	.022	.448	.654
**Age group**	** ≤ 25**	26–35 36–45 **46–55** 56 +	.138 − .216 ** − .580** .449	.153 .236 **.255** .389	.045 − .044 ** − .110** .052	.903 − .914 − **2.279** 1.155	.367 .361 .023* .249
**Career stage**	**Students**	**Doctors**	− **1.035**	**.160**	** − .382**	− **6.460**	< .001*

^*^ statistically significant (p < .05)

Bold rows indicate significant results

Finally, free-text responses to the two questions eliciting barriers to seeking support at all and to seeking earlier support were analysed using content analysis, where qualitative responses were grouped and counted according to the concepts they conveyed [30]. Microsoft Excel was used to perform the analysis grouping relevant phrases into themed codes. Initial coding which emerged from the text was done by ME and these were discussed and refined by all three authors. Results were combined across the two questions due to overlapping themes, and summarised in a frequency table (Table 4) to show how help-seeking for financial problems may be improved.

**Table 4:** What would improve help-seeking?

Response	N
Better awareness and understanding of available support—including improved signposting and advertising, clearer understanding of eligibility, more transparent/straightforward processes, current barriers include assuming seeking help would be unsuccessful/ assumed ineligibility	63
Reduced stigma—make financial problems a more widely discussed subject, current barriers include pride, shame, expectations doctors are ‘wealthy’ and cannot be struggling financially, did not want others to find out, e.g. within social circle, educational body—worried about it affecting progression	27
Improved financial support and resources offered—including better funding for medics, less stringent criteria for support, interest free/ low interest short-term loans that are less daunting to access	16
Earlier realisation that they were in financial difficulty or that help would be useful	8
Better transparency and awareness of the costs of education and training—including exams	3
Worse financial position—would only seek help in a ‘crisis situation’	2
Anonymous support systems—complete belief that help-seeking would be confidential	2
Better assistance with accessing support—including assistance with filling forms, ‘battling’ departments, current barriers include busy with exams / reluctance to commence a time and effort-consuming process	4
Employment—being allowed to take up a job while at university to supplement income	1
Other—unsure, not specified, nothing/ not possible (financial difficulty was not predicted; not necessary due to financial ‘safety net’ of parents; did seek early support; did not require support; felt they were responsibility so should deal with it themselves)	19

## Results

The demographic characteristics of the 442 responders are displayed in Table 1. Participants were predominantly female (69.0%, 305); heterosexual (82.1%, 363); single (47.3%, 209); from a white ethnic background (69.5%, 307) and UK trained (total 87.8%). Most participants did not have a disability (85.1%, 376) nor caring responsibilities (77.4%, 342). The median age was 19 (interquartile range 13). Nearly half of participants were medical students (45.2%, 200); nearly a quarter were doctors in specialty training (23.1%, 102), with the remainder of the cohort made up of consultants (12.2%, 54), general practitioners (GPs) (7.0%, 31), foundation level (3.4%, 15) and other doctors (8.8%, 39).

### Experiences of financial worry and difficulty

In total, 372 out of 442 (84.2%) responded “yes” to having ever experienced financial worry, 68 (15.4%) reported never having worried and 2 (0.5%) preferred not to say. In total, 148 out of 442 (33.5%) responded “yes” to having ever experienced financial difficulty, 286 (64.7%) reported never being in financial difficulty, with 8 (1.8%) preferring not to say.

Table 2 demonstrates that participants who identified as LGBTQ + had greater odds of having ever worried about their financial situation compared to those identifying as heterosexual (OR = 3.54, *p* = 0.039). Additionally, the odds of non-UK PMQ participants experiencing financial difficulty were two times greater compared to UK-PMQ respondents (*p* = 0.039). All other results were non-significant.

A summary of the significant exploratory Chi-square analysis results for ‘Ever worried about / experienced financial difficulty?’ (Appendix A) is included here, as a tentative indicator of relationships that may have been identified had all 9 demographic characteristics been included in the regression analysis.

In the group of those who experienced financial worries, there was a significantly higher percentage of LGBTQ + respondents (94.7%) compared to heterosexual respondents (82.6%; x^2^ (1) = 5.469, *p* = 0.019); respondents currently in a relationship (88.2%) compared to single participants (80.8%; x^2^ (1) = 4.448, *p* = 0.035); white respondents (86.6%) compared to those from ethnic minority backgrounds (78.9%; x^2^ (1) = 4.044, *p* = 0.044); respondents with a disability (96.6%) compared to those without (82.7%; x^2^ (1) = 7.404, *p* = 0.007); those with caring responsibilities (91.2%) compared to those without (82.4%; x^2^ (1) = 4.198, *p* = 0.040) and finally, those in the 26–35 age bracket (39.9%) compared to all other age brackets (x^2^ (4) = 14.659, *p* = 0.005).

In the group of those who experience financial difficulties, there were a higher percentage of respondents with a disability (53.4%) than those without a disability (30.4%; x^2^ (1) = 11.899, *p* < 0.001); those with caring responsibilities (47.2%) compared to those without (30.4%; x^2^ (1) = 12.450, *p* = 0.014); and finally, respondents within the 26–35 age bracket (47.1%) compared to all other age brackets, (x^2^ (4) = 12.450, *p* = 0.014).

### Intentions to seek help

Table 3 shows that there were no significant differences between demographic groups when all sources of help were analysed collectively, and when personal sources of help were analysed. However, participants with an overseas PMQ were more likely to seek external sources of help than those with a UK-PMQ (*β* = 0.099, *p* = 0.041). Similarly, those in the 36–45 age bracket were more likely to seek external sources of help than those in the ≤ 25 age bracket (*β* = 0.126, *p* = 0.016), and doctors were more likely to seek external support than students (*β* = 0.170, *p* = 0.008). Regarding education and workplace sources of support, those without a disability were more likely to seek help compared to those with a disability (β = − 0.093**,** p = 0.037). Additionally, those with a UK-PMQ were more likely to seek this help compared to participants with an overseas PMQ (*β* = − 0.108, *p* = 0.017). Participants in the ≤ 25 age bracket were also more likely to seek this help compared to older participants 46–55 (β = − 0.110, *p* = 0.023) and finally, students were more likely to seek this support compared to doctors (*β* = − 0.382, *p* < 0.001).

Table 4 displays the frequencies resulting from content analysis of combined answers to the following two questions: ‘What would have helped you to seek support or seek earlier support?’ answered by 105 respondents, and ‘If you did not seek help, why not?’ answered by 30 respondents. The most common response stemmed around improving awareness and understanding available support (*N* = 63), followed by reducing stigma (*N* = 27). These problems were exemplified by one respondent*:*

*‘Better awareness of what resources were available. I also felt ashamed of my situation as it seemed most of my friends had money, so caring less about what people might think would also have helped.’ [Female doctor]*

The third commonest answer was improved financial support, increased resources and funding to subsidise education and training costs:

*‘I don’t think the cost associated with training is recognised by trusts or deaneries—people moving for their work and struggling to find affordable places to live, plus commuting. Recognition of this and support offered locally may help alleviate people getting in financial trouble, I may have accessed this.’ [Female doctor]*

These costs, and the need for more robust support, were recognised at both a postgraduate and undergraduate level:

*‘One of the issues I have is that when looking at financial help available from my university, it states that you have to be overdrawn on your current account to qualify for help, which for a regular 19-year-old student, might be reasonable. But as a mature student with a mortgage, outstanding finance payments, repayments on existing loans, life insurance, medical insurance, childcare costs, etc., it just is not a reasonable course of action, as all of the above would wipe out my entire agreed overdraft swiftly. So I would effectively have to demonstrate awful financial acumen to be eligible for help. In my opinion, such help should be contingent on working on financial planning and projections.’ [Male student]*

## Discussion

To the author’s knowledge this is the first study investigating the experiences of financial hardship and help-seeking behaviour of medics across different demographic backgrounds. With our findings, we seek to improve knowledge on how the increasingly diverse medical workforce may be better supported with preventative and responsive measures to financial difficulty, which impacts staff well-being, performance, and retention.

Overall, experiences of financial problems were common, with over three-quarters of participants reporting ever experiencing financial worry (84.2%) and over a third reporting ever experiencing financial difficulty (33.5%). This shows the importance of investigating financial insecurities in medicine and the need for improved support. Our study revealed that certain groups are more likely to worry or experience financial difficulties. First, those identifying as LGBTQ + had 3.54 greater odds to worry about their financial situation. Although the confidence interval for this finding is wide and this result should be interpreted with caution, current literature does discuss potential mechanisms through which students’ sexual orientation could contribute to financial difficulties. A recent UK survey of nearly 2500 participants examined the experiences of LGBTQ + medical students and doctors [31]. While financial difficulty was not discussed explicitly, they were shown to face discrimination, phobia and bias in the education and training systems, ranging from subtler negative interactions such as undermining and questioning of competence to overt bullying, harassment. Negative impacts on mental health (including stress, depression and anxiety), loss of motivation in their studies or work, and considering leaving or leaving their job were also reported [31]. Similarly, LGBTQ + medical students have been found to experience higher levels of social stressors and social isolation [32]. These repercussions demonstrate potential mechanisms that may contribute to vulnerability to financial hardship among the LGBTQ + group, including reduced support from professional networks, barriers to career progression, and time out of work. It is important to highlight though that our finding should be taken with caution considering wide confidence intervals and future research on the LGBTQ + medical profession should investigate this topic further by ensure financial hardship and possible contributors are explored.

Second, our study showed that having a non-UK PMQ made a medic twice as likely to experience financial difficulty. Overseas medics have been identified as vulnerable group for financial challenges in previous publications [33]. Causes of financial challenges may include shouldering higher student fees and set-up expenses when moving to the UK [28], insufficient access to financial support either in the UK [34] or from their home country [35], and requirements to financially support family overseas.

Our exploratory analyses also found that financial challenges may disproportionately affect those with a disability (53.4% vs 30.45 without a disability) or caring responsibilities (47.2% versus 30.4%) may be particularly vulnerable to financial challenges. We could not include these groups in further analyses. However, current evidence does suggest these doctors are likely to be particularly vulnerable to financial difficulty. Reasons for those with a disability include having to work less-than-full-time [36], barriers to working and accessing the financial support they qualify for [37]. For carers, limited time for paid work and costs towards those they support may lead to financial strain [38]. Also, undergraduate medicine cannot be studied part time and ‘flexible working’ policies that may support carers in non-clinical settings are not existent to the same extent in the current UK healthcare system. We suggest these groups as another important focus for future research and practice in understanding and supporting financial hardship among the medical profession, including increasing flexibility within the delivery of education and training to ensure these groups are not disadvantaged in accessing opportunities, and reduce differential attainment.

Our analysis of intentions to seek help found that medics were more likely to seek education and workplace sources of support (professional organisations, workplace, student loan) if they did not have a disability, had a UK-PMQ, were a student or were in the ≤ 25 age bracket. Additionally, our study found that respondents with an overseas PMQ were more likely to seek external sources of help (bank, charity, loan, government) than their UK-PMQ counterparts, as were doctors in the 36–45 age bracket compared to those under 25. These findings suggest that current internal/ local support structures within education and workplace systems may be underutilised by medics with disabilities, non-UK graduates, and medics in postgraduate settings more broadly. Indeed, in a recent UK study on doctors and medical students with disabilities, accessing additional funding and support were described as burdensome and lengthy processes, with insufficient employer awareness obstructive to timely processing of applications [36]. For overseas students and international medical graduates, unfamiliarity with UK systems and reduced inclination to disclose personal information as a result of negative previous experiences with organisations pertaining to immigration [28].

Furthermore, one of the three most recommended improvements to facilitate help-seeking reported by participants in this study was high postgraduate training costs resulting in a need for improved financial support and funding for medics. It is known that medicine is an expensive career to pursue both at undergraduate and postgraduate level [5, 39], a problem worsened by doctors’ pay failing to keep up with rising inflation. Improved awareness and understanding of available support and reduced stigma were the two other most common responses for removing barriers to help-seeking for financial difficulty, which work hand in hand. While stigma is an established problem surrounding financial difficulty across the population [40], it may be particularly problematic among a profession commonly misconstrued as being financially secure, especially deniable in the earlier years of studying and training [3, 7]. Stigma surrounding financial difficulty may exacerbate the problem by delaying or denying help-seeking, as well as worsening the mental health of those affected [21].

Our study is not without limitations. We were unable to calculate the response rate to our questionnaire. The three groups of resources for financial support were constructed by the authors to provide more depth to the analysis. Although the authors consider the results to be relevant, reliability analysis showed the performance of these three constructs were lower than desired and so the results based on these groupings should be interpreted with caution. We could not analyse sub-groups within ‘ethnic minority’ status due to small numbers (we already see wide confidence intervals). We were also unable to include all demographic groups in all analyses due to the nature of the original questionnaire from which the current study’s data were derived. Furthermore, the questionnaire did not survey socioeconomic status, which we recommend is included in future studies assessing financial hardship among medics and evaluate support systems, as it is a potential confounder and those from low-income backgrounds may be particularly vulnerable, for example during the latter years of medical school when governmental financial support is insufficient [4], and during the transition to foundation training before getting paid [21]. Future research should also explore potential differences between the two groups, doctors and medical students.

## Conclusion

Our study has highlighted that LGBTQ + medics and those with a non-UK PMQ may be particularly vulnerable to financial problems, with tentative findings indicating further research and practice should focus on medics with a disability and those with caring roles as two additional groups potentially vulnerable to financial problems. Additionally, current education and workplace mechanisms of financial support may be underutilised by medics with disabilities, non-UK graduates, and medics in postgraduate settings more broadly. Local and national institutions should improve access to financial support: the most sought improvements included better awareness and understanding of resources, more robust financial support and funding for medics; and efforts to reduce stigma of financial hardship in the medical profession.

## Supplementary Information

Additional file 1.

## Data Availability

The datasets generated and analysed during the current study are not publicly available as this is not covered by participant consent.

## Abbreviations

ANOVA: Analysis of variance
LGBTQ +: Lesbian, gay, bisexual, transgender, queer or questioning persons and more
NHS: National Health Service
PMQ: Primary Medical Qualification
RMBF: Royal Medical Benevolent Fund
SPSS: Statistical Package for the Social Sciences
UK: United Kingdom

## Footnotes

1 Foundation training is a 2-year work-based training programme which medical student enter after graduation. This is a general training before undertaking specialty/general practice training.

2 UK undergraduate medical training takes four to six years in the UK and is charged a tuition fee.
