# Supplementary material for: Exploring financial difficulty and help-seeking behaviour among medics in the United Kingdom: a cross-sectional survey
Source: Hum Resour Health. 2025 Aug 28;23:49. doi: 10.1186/s12960-025-01008-0 (PMC12395633; doi:10.1186/s12960-025-01008-0)
Supplement: Supplementary file 1 — Additional file 1. [file 12960_2025_1008_MOESM1_ESM.docx]

Appendix A . Chi-square tests of independence analysing financial worries and difficulties among different demographic groups.

| **Group** | **Sub-group** | **Have you ever worried about your financial situation?** | | | **Have you ever experienced financial difficulties?** | | |
| --- | --- | --- | --- | --- | --- | --- | --- |
|  |  | **Yes (%)** | **No (%)** | **X, df**  **(p)** | **Yes (%)** | **No (%)** | **X, df**  **(p)** |
| Gender | Male | 112 (83.6) | 22 (16.4) | .117, 1 (.732) | 46 (34.8) | 86 (65.2) | .081, 1 (.777) |
|  | Female | 258 (84.9) | 46 (15.1) |  | 100 (33.4) | 199 (66.6) |  |
| Sexuality | Heterosexual | **299 (82.6)** | **63 (17.4)** | **5.469, 1 (.019*)** | 118 (33.2) | 237 (66.8) | .000, 1 (.989) |
|  | LGBTQ+ | **54 (94.7)** | **3 (5.3)** |  | 19 (33.3) | 38 (66.7) |  |
| Relationship status | Single | **181 (80.8)** | **43 (19.2)** | **4.448, 1 (.035*)** | 72 (32.9) | 147 (67.1) | .305, 1 (.581) |
|  | Relationship | **186 (88.2)** | **25 (11.8)** |  | 74 (35.4) | 135 (64.6) |  |
| Ethnicity | White | **266 (86.6)** | **41 (13.4)** | **4.044, 1 (.044*)** | 107 (35.2) | 197 (64.8) | .642, 1 (.423) |
|  | Ethnic minority | **97 (78.9)** | **26 (21.1)** |  | 37 (31.1) | 82 (68.9) |  |
| Disability | Disability | **56 (96.6)** | **2 (3.4)** | **7.404, 1 (.007*)** | **31 (53.4)** | **27 (46.6)** | **11.899, 1 (<.001*)** |
|  | No disability | **310 (82.7)** | **65 (17.3)** |  | **112 (30.4)** | **256 (69.6)** |  |
| Carer status | Carer | **83 (91.2)** | **8 (8.8)** | **4.198, 1 (.040*)** | **42 (47.2)** | **47 (52.8)** | **8.901, 1 (.003*)** |
|  | Not a carer | **281 (82.4)** | **60 (17.6)** |  | **102 (30.4)** | **234 (69.6)** |  |
| PMQ | UK | 329 (85.2) | 57 (14.8) | 1.586, 1 (.208) | 126 (33.0) | 256 (67.0) | 1.884, 1 (.170) |
|  | Non-UK | 40 (78.4) | 11 (21.6) |  | 21 (42.9) | 28 (57.1) |  |
| Career stage | Student | 165 (83.3) | 33 (16.7) | 7.154, 5  (.209) | 65 (33.5) | 129 (66.5) | 8.005, 5  (.156) |
|  | Foundation | 10 (66.7) | 5 (33.3) |  | 2 (13.3) | 13 (86.7) |  |
|  | Specialty tr. | 91 (89.2) | 11 (10.8) |  | 41 (40.6) | 60 (59.4) |  |
|  | GP | 28 (90.3) | 3 (9.68) |  | 12 (40.0) | 18 (60.0) |  |
|  | Consultant | 46 (85.2) | 8 (14.8) |  | 13 (24.1) | 41 (75.9) |  |
|  | Other | 31 (79.5) | 8 (20.5) |  | 15 (38.5) | 24 (61.5) |  |
| **Age** | **≤25** | **83 (32.2)** | **26 (59.1)** | **14.659, 4 (.005*)** | **24 (23.5)** | **85 (42.9)** | **12.450, 4 (.014*)** |
|  | **26-35** | **103 (39.9)** | **10 (22.7)** |  | **48 (47.1)** | **65 (32.8)** |  |
|  | **36-45** | **35 (13.6)** | **2 (4.5)** |  | **15 (14.7)** | **21 (10.6)** |  |
|  | **46-55** | **28 (10.9)** | **3 (6.8)** |  | **12 (11.8)** | **18 (9.1)** |  |
|  | **56+** | **9 (3.5)** | **3 (6.8)** |  | **3 (2.9)** | **9 (4.5)** |  |

* = statistically significant (p<.05)

**Experienced financial worry:** In total, 372 out of 442 (84.2%) responded “yes” to having ever experienced financial worry, 68 (15.4%) reported never having worried and 2 (.5%) preferred not to say. The following significant differences between groups having ever experienced financial worries were found. There was a higher percentage of LGBTQ+ respondents (94.7%) compared to heterosexual respondents (82.6%,) x^2^ (1) = 5.469, p=.019; respondents currently in a relationship (88.2%) compared to single participants (80.8%), x^2^ (1) = 4.448, p=.035; white respondents (86.6%) compared to those from ethnic minority backgrounds (78.9%), x^2^ (1) = 4.044, p=.044; respondents with a disability (96.6%) compared to those without (82.7%), x^2^ (1) = 7.404, p=.007; those with caring responsibilities (91.2%) compared to those without (82.4%), x^2^ (1) = 4.198, p=.040; and finally, those in the 26-35 age bracket (39.9%) compared to all other age brackets, x^2^ (4) = 14.659, p=.005.

**Experienced financial difficulty:** In total, 148 out of 442 (33.5%) responded “yes” to having ever experienced financial difficulty, 286 (64.7%) reported never being in financial difficulty, with 8 (1.8%) preferring not to say. The following significant differences between groups having ever experienced financial difficulty were found. There were a higher percentage of respondents with a disability (53.4%) than those without a disability (30.4%), x^2^ (1) = 11.899, p<.001; those with caring responsibilities (47.2%) compared to those without (30.4%), x^2^ (1) = 12.450, p=.014; and finally, respondents within the 26-35 age bracket (47.1%) compared to all other age brackets, x^2^ (4) = 12.450, p=.014.

Appendix B. Likelihood of seeking personal sources of support sought for financial difficulty among demographic groups using Chi-square analysis.

|  |  | **Likely to seek personal source of support** | | | | | | | | | | | |
| --- | --- | --- | --- | --- | --- | --- | --- | --- | --- | --- | --- | --- | --- |
| **Group** | **Sub-group** | **Partner** | | | | **Family** | | | | **Friends** | | | |
|  |  | **Yes**  **(%)** | **No**  **(%)** | **Neut**  **(%)** | **X, df**  **(p)** | **Yes**  **(%)** | **No**  **(%)** | **Neut**  **(%)** | **X, df**  **(p)** | **Yes**  **(%)** | **No**  **(%)** | **Neut**  **(%)** | **X, df (p)** |
| Gender | Male | 95 (71.4) | 27 (20.3) | 11  (8.3) | 1.456, 2  (.483) | 106  (80.3) | 20  (15.2) | 6  (4.5) | .336, 2  (.845) | 35  (26.5) | 85  (64.4) | 12  (9.1) | 1.142, 2, (.565) |
|  | Female | 223 (73.4) | 49 (16.1) | 32  (10.5) |  | 250  (82.0) | 40  (13.1) | 15  (4.9) |  | 67  (22.1) | 33  (10.9) | 203  (67.0) |  |
| Sexuality | Hetero-  sexual | 271  (75.1) | 57  (15.8) | 33  (9.1) | 3.688, 2,  (.158) | 295  (81.7) | 48  (29.8) | 18  (11.2) | .248, 2,  (.883) | 83  (23.1) | 238  (66.1) | 39  (10.8) | .341, 2, (.843) |
|  | LGBTQ+ | 36  (63.2) | 7  (12.3) | 14  (24.6) |  | 47  (82.5) | 8  (14.0) | 2  (3.5) |  | 11  (19.6) | 39  (69.6) | 6  (10.7) |  |
| **Relationship status** | **Single** | **139 (62.3)** | **53**  **(23.8)** | **31**  **(13.9)** | **24.210, 2, (<.001*)** | **193**  **(86.5)** | **20**  **(9.0)** | **10**  **(4.5)** | **9.990, 2, (.007*)** | 59  (26.6) | 140  (63.1) | 23  (10.4) | 3.035, 2, (.219) |
|  | **Relation-ship** | **176 (83.4)** | **22**  **(10.4)** | **13**  **(6.2)** |  | **160**  **(75.8)** | **41**  **(19.4)** | **10**  **(4.7)** |  | 41  (19.5) | 146  (69.5) | 23  (11.0) |  |
| Ethnicity | White | 218  (71.2) | 57  (18.6) | 31  (10.1) | .609, 2,  (.738) | 247  (81.0) | 42  (13.8) | 16  (5.2) | .283, 2  (.868) | 63  (20.7) | 209  (68.8) | 32  (10.5) | 4.446, 2, (.108) |
|  | Ethnic  minority | 91  (74.0) | 19  (15.4) | 13  (10.6) |  | 102  (82.3) | 17  (13.7) | 5  (4.0) |  | 37  (30.1) | 73  (59.3) | 13  (10.6) |  |
| Disability  status | Disability | 36  (62.1) | 15  (25.9) | 7  (12.1) | 3.902, 2,  (.142) | 43  (74.1) | 12  (20.7) | 3  (5.2) | 2.661, 2  (.264) | 17  (29.8) | 36  (63.2) | 4  (7.0) | 2.062, 2 (.357) |
|  | No disability | 277  (74.1) | 61  (16.3) | 36  (9.6) |  | 308  (82.4) | 48  (12.8) | 18  (4.8) |  | 83  (22.3) | 249  (66.8) | 41  (11.0) |  |
| **Carer**  **status** | **Carer** | 71  (78.0) | 13  (14.3) | 7  (7.7) | 1.710, 2,  (.425) | **65**  **(71.4)** | **22**  **(24.2)** | **4**  **(4.4)** | **10.148, 2, (.006*)** | 20  (22.5) | 60  (67.4) | 9  (10.1) | .106, 2, (.949) |
|  | **Not a carer** | 242  (71.2) | 62  (18.2) | 36  (10.6) |  | **286**  **(84.1)** | **38**  **(11.2)** | **16**  **(4.7)** |  | 81  (23.8) | 223  (65.6) | 36  (10.6) |  |
| PMQ | UK | 276  (71.5) | 72  (18.7) | 38  (9.8) | 3.682, 2,  (.159) | 316  (81.7) | 53  (13.7) | 18  (4.7) | .186, 2,  (.911) | 88  (22.9) | 253  (65.9) | 43  (11.2) | 2.727, 2, (.256) |
|  | Non-UK | 41  (80.4) | 4  (7.8) | 6  (11.8) |  | 40  (80.0) | 7  (14.0) | 3  (6.0) |  | 14  (28.0) | 34  (68.0) | 2  (4.0) |  |
| **Career stage^1^** | **Student** | **124**  **(62.6)** | **51**  **(25.8)** | **23**  **(11.6)** | **19.593, 2, (<.001*)** | **167**  **(84.3)** | **19**  **(9.6)** | **12**  **(6.1)** | **6.432, 2, (.040*)** | 46  (23.5) | 134  (68.4) | 16  (8.2) | 1.840, 2, (.398) |
|  | **Doctor** | **194**  **(80.5)** | **26**  **(10.8)** | **21**  **(8.7)** |  | **190**  **(78.8)** | **42**  **(17.4)** | **9**  **(3.7)** |  | 56  (23.3) | 155  (64.6) | 29  (12.1) |  |
| **Age** | **≤25** | **69**  **(62.7)** | **27**  **(24.5)** | **14**  **(12.7)** | **18.866, 8, (.016*)** | **100**  **(91.7)** | **6**  **(5.5)** | **3**  **(2.8)** | **26.800, 8, (<.001*)** | 32  (29.6) | 71  (65.7) | 5  (4.6) | 13.333, 8, (.101) |
|  | **26-35** | **91**  **(80.5)** | **10**  **(8.8)** | **12**  **(10.6)** |  | **90**  **(79.6)** | **17**  **(15.0)** | **6**  **(5.3)** |  | 24  (21.4) | 71  (63.4) | 17  (15.2) |  |
|  | **36-45** | **24**  **(64.9)** | **10**  **(27.0)** | **3**  **(8.1)** |  | **30**  **(81.1)** | **3**  **(8.1)** | **4**  **(10.8)** |  | 8  (21.6) | 22  (59.5) | 7  (18.9) |  |
|  | **46-55** | **26**  **(83.9)** | **4**  **(12.9)** | **1**  **(3.2)** |  | **20**  **(64.5)** | **11**  **(35.5)** | **0**  **(0.0)** |  | 6  (19.4) | 23  (74.2) | 2  (6.5) |  |
|  | **56+** | **8**  **(66.7)** | **1**  **(8.3)** | **3**  **(25.0)** |  | **10**  **(83.3)** | **1**  **(8.3)** | **1**  **(8.3)** |  | 2  (16.7) | 7  (58.3) | 3  (25.0) |  |

Yes = respondents voting ‘extremely likely’, ‘very likely’ or ‘likely’

No = respondents voting ‘extremely unlikely’, ‘very unlikely’ or ‘unlikely’

Neut = respondents voting ‘neutral’

1 = condensed sub-groups (students/doctors) due to expected count assumption violated when all career stages inputted

* = statistically significant (p<.05)

**Partner**: A significantly higher percentage of respondents currently in a relationship (83.4%) responded as likely to seek partner support for financial difficulty than single participants (62.3%), as expected, x^2^ (2) = 24.210, p <.001. Additionally, a higher proportion of those in the 46-55 age bracket (83.9%) responded as likely to seek help from a partner compared to all other age brackets, x^2^ (8) = 18.866, p=.016. A higher percentage of doctors (80.5%) were more likely than students (62.6%) to seek support from partners, x^2^ (2) = 19.593, p<.001.

**Family**: A higher percentage of single participants (86.5%) responded as likely to seek support from family compared to those in a relationship (75.8%), x^2^ (2) = 9.990, p=.007; a higher percentage of those in the ≤25 age bracket (91.7%) compared to those from all other age brackets, x^2^ (8) = 26.800, p<.001; a higher percentage of those without caring roles (84.1%) compared to carers (71.4%), x^2^ (2) = 10.148, p=.006); and a higher proportion of students (84.3%) than doctors (78.8%), x^2^ (2) = 6.432, p=.040.

**Friends**: No significant differences among groups found.

Appendix C. Likelihood of seeking education / workplace-based sources of support sought for financial difficulty among demographic groups using Chi-square analysis.

|  |  | **Likely to seek education/ workplace source of support** | | | | | | | | | | | |
| --- | --- | --- | --- | --- | --- | --- | --- | --- | --- | --- | --- | --- | --- |
| **Group** | **Sub-group** | **Workplace** | | | | **Student Loan** | | | | **Professional Organisations** | | | |
|  |  | **Yes**  **(%)** | **No**  **(%)** | **Neut**  **(%)** | **X, df**  **(p)** | **Yes**  **(%)** | **No**  **(%)** | **Neut**  **(%)** | **X, df**  **(p)** | **Yes**  **(%)** | **No**  **(%)** | **Neut**  **(%)** | **X, df (p)** |
| **Gender** | Male | 52  (39.1) | 61  (45.9) | 20  (15.0) | 1.662, 2,  (.436) | **38**  **(28.8)** | **76 (57.6)** | **18**  **(13.6)** | **11.083, 2, (.004*)** | 46  (34.6) | 55  (41.4) | 32  (24.1) | .337, 2, (.845) |
|  | Female | 132  (43.6) | 119  (39.3) | 52  (17.2) |  | **126**  **(41.3)** | **123**  **(40.3)** | **56**  **(18.4)** |  | 113  (85.0) | 125  (37.0) | 67  (22.0) |  |
| Sexuality | Hetero-  sexual | 148  (41.1) | 150  (41.7) | 62  (17.2) | .411, 2 (.814) | 129  (35.7) | 165  (45.7) | 67  (18.6) | 2.533, 2, (.282) | 138  (38.1) | 149  (41.2) | 75  (20.7) | 4.507, 2, (.105) |
|  | LGBTQ+ | 26  (45.6) | 22  (38.6) | 9  (15.8) |  | 26  (45.6) | 24  (45.6) | 7  (12.3) |  | 15  (26.3) | 24  (42.1) | 18  (31.6) |  |
| **Relationship status** | **Single** | **115**  **(51.6)** | **70**  **(31.4)** | **38**  **(17.0)** | **20.033, 2, (<.001*)** | **109**  **(48.7)** | **83**  **(37.1)** | **32**  **(14.3)** | **23.296, 2, (<.001*)** | 86  (38.4) | 90  (40.2) | 48  (21.4) | .675, 2, (.714) |
|  | **Relation-ship** | **68**  **(32.4)** | **108**  **(51.4)** | **34**  **(16.2)** |  | **55**  **(26.2)** | **113**  **(53.8)** | **42**  **(20)** |  | 73  (34.6) | 90  (42.7) | 48  (22.7) |  |
| Ethnicity | White | 122  (40.0) | 134  (43.3) | 49  (16.1) | 2.949, 2 (.229) | 119  (39.0) | 132  (43.3) | 54  (17.7) | 1.630, 2, (.443) | 107  (35.0) | 125  (40.8) | 74  (24.2) | 1.408, 2, (.495) |
|  | Ethnic  minority | 58  (47.2) | 43  (35.0) | 22  (17.9) |  | 42  (33.9) | 62  (50) | 20  (16.1) |  | 49  (39.5) | 51  (41.1) | 24  (19.4) |  |
| **Disability**  **status** | **Disability** | **14**  **(24.6)** | **30**  **(52.6)** | **13**  **(22.8)** | **8.497, 2, (.014*)** | **16**  **(27.6)** | **26**  **(44.8)** | **16**  **(27.6)** | **6.431, 2, (.040*)** | 17  (29.3) | 24  (41.4) | 17  (29.3) | 2.319, 2, (.314) |
|  | **No disability** | **168**  **(44.9)** | **148**  **(39.6)** | **58**  **(15.5)** |  | **148**  **(39.6)** | **169**  **(45.2)** | **57**  **(15.2)** |  | 140  (37.3) | 155  (41.3) | 80  (21.3) |  |
| **Carer**  **status** | **Carer** | **23**  **(25.3)** | **51**  **(56.0)** | **17**  **(18.7)** | **15.040, 2, (<.001*)** | **22**  **(24.2)** | **54**  **(59.3)** | **15**  **(16.5)** | **10.838, 2 (.004*)** | 31  (8.3) | 36  (34.1) | 24  (26.4) | .791, 2 (.673) |
|  | **Not a carer** | **160**  **(47.2)** | **124**  **(36.6)** | **55**  **(16.2)** |  | **140**  **(41.2)** | **140**  **(41.2)** | **60**  **(17.6)** |  | 125  (36.7) | 141  (41.3) | 75  (22.0) |  |
| PMQ | UK | 165  (43.0) | 157  (40.9) | 62  (16.1) | .267, 2 (.875) | 148  (38.4) | 167  (43.4) | 70  (18.2) | 5.825, 2, (.054) | 139  (36.0) | 159  (41.2) | 88  (22.8) | .332, 2, (.847) |
|  | Non-UK | 20  (39.2) | 22  (43.1) | 9  (17.6) |  | 15  (29.4) | 31  (60.8) | 5  (9.8) |  | 20  (39.2) | 21  (41.2) | 10  (19.6) |  |
| **Career stage** | **Student** | **122**  **(61.6)** | **51**  **(25.8)** | **25**  **(12.6)** | **69.387, 10, (<.001*)** | **121**  **(61.1)** | **54**  **(27.3)** | **23**  **(11.6)** | **94.496, 10, (<.001*)** | 75  (37.9) | 83  (41.9) | 40  (20.2) | 13.227, 10, (.211) |
|  | **FY** | **7**  **(46.7)** | **4**  **(26.7)** | **4**  **(26.7)** |  | **3**  **(20.0)** | **10**  **(66.7)** | **2**  **(13.3)** |  | 7  (46.7) | 7  (46.7) | 1  (6.7) |  |
|  | **Specialty** | **31**  **(31.0)** | **47**  **(47.0)** | **22**  **(22.0)** |  | **24**  **(23.5)** | **58**  **(56.9)** | **20**  **(19.6)** |  | 41  (40.2) | 32  (31.4) | 29  (28.4) |  |
|  | **GP** | **3**  **(9.7)** | **22**  **(71.0)** | **6**  **(19.4)** |  | **4**  **(12.9)** | **21**  **(67.7)** | **6**  **(19.4)** |  | 11  (35.5) | 13  (41.9) | 7  (22.6) |  |
|  | **Cons** | **11**  **(20.4)** | **33**  **(61.1)** | **10**  **(18.5)** |  | **3**  **(5.7)** | **34**  **(64.2)** | **16**  **(30.2)** |  | 18  (33.3) | 23  (42.6) | 13  (24.1) |  |
|  | **Other** | **11**  **(28.2)** | **23**  **(59.0)** | **5**  **(12.8)** |  | **9**  **(23.1)** | **22**  **(56.4)** | **8**  **(20.5)** |  | 8  (20.5) | 23  (59.0) | 8  (20.5) |  |
| **Age** | ≤**25** | **66**  **(60.6)** | **23**  **(21.1)** | **20**  **(18.3)** | **42.824, 8, (<.001*)** | **61**  **(56.0)** | **37**  **(33.9)** | **11**  **(10.1)** | **47.464, 8 (<.001*)** | 45  (41.3) | 45  (41.3) | 19  (17.4) | 6.418, 8, (.600) |
|  | **26-35** | **44**  **(39.3)** | **46**  **(41.1)** | **22**  **(19.6)** |  | **36**  **(31.9)** | **53**  **(46.9)** | **24**  **(21.2)** |  | 39  (34.5) | 39  (34.5) | 35  (31.0) |  |
|  | **36-45** | **6**  **(16.7)** | **23**  **(63.9)** | **7**  **(19.4)** |  | **7**  **(18.9)** | **22**  **(59.5)** | **8**  **(21.6)** |  | 13  (35.1) | 16  (43.2) | 8  (21.6) |  |
|  | **46-55** | **4**  **(12.9)** | **21**  **(67.7)** | **6**  **(19.4)** |  | **0**  **(0.0)** | **25**  **(80.6)** | **6**  **(19.4)** |  | 12  (38.7) | 12  (38.7) | 7  (22.6) |  |
|  | **56+** | **4**  **(33.3)** | **6**  **(50.0)** | **2**  **(16.7)** |  | **4**  **(36.4)** | **3**  **(27.3)** | **4**  **(36.4)** |  | 5  (41.7) | 5  (41.7) | 2  (16.7) |  |

Yes = respondents voting ‘extremely likely’, ‘very likely’ or ‘likely’

No = respondents voting ‘extremely unlikely’, ‘very unlikely’ or ‘unlikely’

Neut = respondents voting ‘neutral’

* = statistically significant (p<.05)

**Workplace:** A significantly higher percentage of single medics responded likely to seek workplace-based support (51.6%) than those in a relationship (32.4%), x^2^ (2) = 20.033, p <.001. This finding was also existent for those without a disability (44.9%) compared to those with a disability (24.6%), x^2^ (2) = 8.497, p=.014; with over half of medics with a disability stating they would not seek support from the workplace (52.6%). Similarly, a higher proportion of participants without a caring role responded likely to seek workplace-based support (47.2%) than carers (25.3%), x^2^ (2) = 15.040, p <.001; with over half of carers reporting being unlikely to engaging with this process (56.0%). Significant differences were also found among different career stages, with a higher percentage of students (61.6%) stating likely to seek workplace-based support compared to all groups of doctors, x^2^ (10) = 69.387, p<.001. This was reflected by age category also, with the highest proportion of ‘likely’ voters within the ≤25 age bracket (60.6%), x^2^ (8) = 42.824, p<.001.

**Student loan:** A higher percentage of females (41.3%) than males (28.8%) responded as likely to opt for student loan support, x^2^ (2) = 11.083, p=.004. This was also the case for single medics (51.6%) compared to those in a relationship (32.4%), x^2^ (2) = 23.296, p <.001; medics without a disability (39.6%) compared to those with a disability (27.6%), x^2^ (2) = 6.431, p=.040; those without caring responsibilities (41.2%) compared to carers (24.2%), x^2^ (2) = 10.838, p=.004. Over half of carers (59.3%) reported being unlikely to seeking this help. A higher percentage of students (61.1%) also selected likely to seek help through student loan support compared to all other career stage groups, x^2^ (10) = 94.496, p<.001, as did medics aged 25 and under (56.0%) compared to other age groups, x^2^ (8) = 47.464, p<.001.

**Professional organisations:** No significant differences among groups found.

Appendix D. Likelihood of seeking external sources of support sought for financial difficulty among demographic groups using Chi-square analysis.

|  |  | **Likely to seek external source of support** | | | | | | | | | | | |
| --- | --- | --- | --- | --- | --- | --- | --- | --- | --- | --- | --- | --- | --- |
| **Group** | **Sub-group** | **Bank** | | | | **Charity** | | | | **Loan** | | | |
|  |  | **Yes**  **(%)** | **No**  **(%)** | **Neut**  **(%)** | **X, df**  **(p)** | **Yes**  **(%)** | **No**  **(%)** | **Neut**  **(%)** | **X, df**  **(p)** | **Yes**  **(%)** | **No**  **(%)** | **Neut**  **(%)** | **X, df (p)** |
| **Gender** | **Male** | 59  (44.7) | 55  (41.7) | 18  (13.6) | 1.925, 2 (.382) | **45**  **(33.8)** | **55**  **(41.4)** | **33**  **(24.8)** | **6.105, 2 (.047*)** | 11  (8.3) | 106  (80.3) | 15  (11.4) | 2.632, 2, (.268) |
|  | **Female** | 153  (50.7) | 119  (39.4) | 30  (9.9) |  | **104**  **(78.2)** | **154**  **(115.8)** | **47**  **(35.3)** |  | 39  (12.8) | 240  (78.9) | 25  (8.2) |  |
| **Sexuality** | **Hetero-**  **sexual** | **181**  **(50.6)** | **136**  **(38.0)** | **41**  **(11.5)** | **8.131, 2 (.017*)** | 128  (35.4) | 168  (40.4) | 66  (18.2) | 1.434, 2 (.488) | 43  (11.9) | 282  (78.3) | 35  (9.7) | 2.399, 2 (.301) |
|  | **LGBTQ+** | **19**  **(33.3)** | **33**  **(57.9)** | **5**  **(8.8)** |  | 16  (28.1) | 31  (54.4) | 10  (17.5) |  | 3  (5.3) | 49  (86.0) | 5  (8.8) |  |
| **Relationship status** | **Single** | **85**  **(38.3)** | **111**  **(50.0)** | **26**  **(11.7)** | **20.479, 2, (<.001*)** | 76  (33.9) | 104  (46.4) | 44  (19.6) | .746, 2, (.689) | 22  (9.8) | 186  (83.0) | 16  (7.1) | 3.531, 2, (.171) |
|  | **Relation-ship** | **124**  **(59.3)** | **63**  **(30.1)** | **22**  **(10.5)** |  | 72  (34.1) | 104  (49.3) | 35  (16.6) |  | 26  (12.4) | 159  (76.1) | 24  (11.5) |  |
| Ethnicity | White | 152  (50.0) | 119  (39.1) | 33  (10.9) | 1.560, 2 (.458) | 105  (34.3) | 144  (47.1) | 57  (18.6) | .229, 2, (.892) | 34  (11.1) | 247  (81.0) | 24  (7.9) | 2.034, 2, (.362) |
|  | Ethnic  minority | 53  (43.4) | 55  (45.1) | 14  (11.5) |  | 42  (33.9) | 61  (49.2) | 21  (16.9) |  | 14  (11.4) | 94  (76.4) | 15  (12.2) |  |
| Disability  status | Disability | 32  (56.1) | 20  (35.1) | 5  (8.8) | 1.566, 2, (.457) | 24  (41.4) | 25  (43.1) | 9  (15.5) | 1.688, 2, (.430) | 6  (10.3) | 49  (84.5) | 3  (5.2) | 1.334, 2, .513 |
|  | No disability | 176  (47.3) | 154  (41.4) | 42  (11.3) |  | 123  (32.8) | 181  (48.3) | 71  (18.9) |  | 42  (11.3) | 295  (79.1) | 36  (9.7) |  |
| **Carer**  **status** | **Carer** | **55**  **(60.4)** | **28**  **(30.8)** | **8**  **(8.8)** | **6.753, 2 (.034*)** | 32  (35.2) | 42  (46.2) | 17  (18.7) | .122, 2, (.941) | 8  (8.8) | 74  (81.3) | 9  (9.9) | .819, 2, (.664) |
|  | **Not a carer** | **152**  **(45.1)** | **145**  **(43.0)** | **40**  **(11.9)** |  | 114  (33.4) | 164  (48.1) | 63  (18.5) |  | 41  (12.1) | 268  (79.1) | 30  (8.8) |  |
| PMQ | UK | 185  (48.4) | 156  (40.8) | 41  (10.7) | .510, 2, (.775) | 127  (32.9) | 190  (49.2) | 69  (17.9) | 3.863, 2, (.145) | **38**  **(9.9)** | **312**  **(81.3)** | **34**  **(8.9)** | **8.529, 2, (.014*)** |
|  | Non-UK | 25  (49.0) | 19  (37.3) | 7  (13.7) |  | 23  (45.1) | 18  (35.3) | 10  (19.6) |  | **12**  **(23.5)** | **34**  **(66.7)** | **5**  **(9.8)** |  |
| **Career stage** | **Student** | **59**  **(30.1)** | **116**  **(59.2)** | **21**  **(10.7)** | **70.092, 10,**  **(<.001*)** | 66  (33.3) | 100  (50.5) | 32  (16.2) | 8.002, 10, (.629) | 18  (9.1) | 170  (85.9) | 10  (5.1) | 15.246, 10, (.123) |
|  | **FY** | **7**  **(46.7)** | **8**  **(53.3)** | **0**  **(0)** |  | 6  (40.0) | 7  (46.7) | 2  (13.3) |  | 2  (13.3) | 11  (73.3) | 2  (13.3) |  |
|  | **Specialty** | **62**  **(60.8)** | **27**  **(26.5)** | **13**  **(12.7)** |  | 41  (40.2) | 41  (40.2) | 20  (19.6) |  | 15  (14.9) | 70  (69.3) | 16  (15.8) |  |
|  | **GP** | **18**  **(58.1)** | **7**  **(22.6)** | **6**  **(19.4)** |  | 9  (29.0) | 18  (56.1) | 4  (12.9) |  | 2  (6.7) | 24  (80.0) | 4  (13.3) |  |
|  | **Cons** | **39**  **(75.0)** | **7**  **(13.5)** | **6**  **(11.5)** |  | 19  (35.2) | 23  (42.6) | 12  (22.2) |  | 8  (14.8) | 41  (75.9) | 5  (9.3) |  |
|  | **Other** | **27**  **(69.2)** | **10**  **(25.6)** | **2**  **(5.1)** |  | 9  (23.1) | 21  (53.8) | 9  (23.1) |  | 5  (12.8) | 31  (79.5) | 3  (7.7) |  |
| **Age** | **≤25** | **33**  **(30.3)** | **67**  **(61.5)** | **9**  **(8.3)** | **48.714, 8, (<.001*)** | 40  (36.7) | 51  (46.8) | 18  (16.5) | 4.476, 8, (.812) | 7  (6.4) | 96  (88.1) | 6  (5.5) | 11.658, 8, (.167) |
|  | **26-35** | **59**  **(52.2)** | **39**  **(34.5)** | **15**  **(13.3)** |  | 35  (31.0) | 55  (48.7) | 23  (20.4) |  | 18  (16.1) | 82  (73.2) | 12  (10.7) |  |
|  | **36-45** | **28**  **(77.8)** | **4**  **(11.1)** | **4**  **(11.1)** |  | 16  (43.2) | 14  (37.8) | 7  (18.9) |  | 5  (13.5) | 28  (75.7) | 4  (10.8) |  |
|  | **46-55** | **23**  **(74.2)** | **4**  **(12.9)** | **4**  **(12.9)** |  | 12  (38.7) | 14  (45.2) | 5  (16.1) |  | 2  (6.5) | 24  (77.4) | 5  (16.1) |  |
|  | **56+** | **7**  **(63.6)** | **2**  **(18.2)** | **2**  **(18.2)** |  | 3  (25.0) | 5  (41.7) | 4  (33.3) |  | 2  (16.7) | 8  (66.7) | 2  (16.7) |  |

Yes = respondents voting ‘extremely likely’, ‘very likely’ or ‘likely’

No = respondents voting ‘extremely unlikely’, ‘very unlikely’ or ‘unlikely’

Neut = respondents voting ‘neutral’

* = statistically significant (p<.05)

|  |  |  |  |  |  |
| --- | --- | --- | --- | --- | --- |
| **Group** | **Sub-group** | **Government** | | | |
|  |  | **Yes**  **(%)** | **No**  **(%)** | **Neut**  **(%)** | **X, df**  **(p)** |
| Gender | Male | 64  (48.1) | 54  (40.6) | 15  (11.3) | 1.734, 2, (.420) |
|  | Female | 138  (45.4) | 117  (38.5) | 49  (16.1) |  |
| Sexuality | Heterosexual | 166  (46.0) | 139  (38.5) | 56  (15.5) | .405, 2 (.817) |
|  | LGBTQ+ | 27  (47.4) | 23  (40.4) | 7  (12.3) |  |
| Relationship status | Single | 110  (49.3) | 77  (34.5) | 36  (16.1) | 4.585, 2 (.101) |
|  | Relationship | 93  (44.1) | 93  (44.1) | 25  (11.8) |  |
| Ethnicity | White | 142  (46.4) | 118  (38.6) | 46  (15.0) | .124, 2 (.940) |
|  | Ethnic  minority | 57  (46.3) | 49  (39.8) | 17  (13.8) |  |
| Disability  status | Disability | 24  (41.4) | 22  (37.9) | 12  (20.7) | 2.259, 2 (.323) |
|  | No disability | 176  (47.1) | 148  (39.6) | 50  (13.4) |  |
| Carer  status | Carer | 43  (47.3) | 34  (37.4) | 14  (15.4) | .199, 2 (.905) |
|  | Not a carer | 157  (46.2) | 135  (39.7) | 48  (14.1) |  |
| PMQ | UK | 179  (46.5) | 153  (39.7) | 53  (13.8) | .407, 2, (.816) |
|  | Non-UK | 25  (49.0) | 18  (35.3) | 8  (15.7) |  |
| Career stage^1^ | Student | 99  (50.3) | 76  (38.6) | 22  (11.2) | 3.634, 2, (.162) |
|  | Doctor | 105  (43.6) | 95  (39.4) | 41  (17.0) |  |
| Age | ≤25 | 53  (48.6) | 38  (34.9) | 18  (16.5) | 9.357, 8, (.313) |
|  | 26-35 | 42  (37.5) | 55  (49.1) | 15  (13.4) |  |
|  | 36-45 | 18  (48.6) | 10  (27.0) | 9  (24.3) |  |
|  | 46-55 | 14  (45.2) | 11  (35.5) | 6  (19.4) |  |
|  | 56+ | 5  (41.7) | 4  (33.3) | 3  (25.0) |  |

Yes = respondents voting ‘extremely likely’, ‘very likely’ or ‘likely’

No = respondents voting ‘extremely unlikely’, ‘very unlikely’ or ‘unlikely’

Neut = respondents voting ‘neutral’

1 = condensed sub-groups (students/doctors) due to expected count assumption violated when all career stages inputted

* = statistically significant (p<.05)

**Bank:** A greater percentage of heterosexual medics (50.6%) felt likely to seek financial support from a bank than those who identified as LGBTQ+ (33.3%), x^2^ (2) = 8.131, p=.017; with over half of the LGBTQ+ group stating they would be unlikely to seek this support (57.9%). A greater proportion selecting likely was also the case for those in a relationship (59.3%) compared to single medics (38.3%), x^2^ (2) = 20.479, p <.001; carers (60.4%) compared to those without a caring role (45.1%), x^2^ (2) = 6.753, p=.034; consultants (75%) compared to any other career-stage category, x^2^ (10) = 70.092, p<.001; and those in the 36-45 age bracket (77.8%) compared to any other age category, x^2^ (8) = 48.714, p<.001. Medics aged 25 and under responded more negatively to requesting help from the bank (61.5% ‘no’).

**Charity:** A greater proportion of females (78.2%) than males (33.8%) felt likely to seek financial help from a charity, x^2^ (2) = 6.105, p=.047.

**Loan:** A higher percentage of those with a non-UK PMQ (23.5%) opted as likely to take out a loan than those qualifying in the UK (9.9%), x^2^ (2) = 8.529, p=.014.

**Government:** No significant differences among groups found.

Appendix E. T-tests comparing average likelihood of seeking any type of help across demographic groups.

| **Groups** | **Subgroups** | **N** | **Mean** | **t** | **df** | **Two-sided p** |
| --- | --- | --- | --- | --- | --- | --- |
| **All sources of help** | | | | | | |
| Gender | Female  Male | 305  133 | 3.9469  3.8243 | 1.397 | 214.152 | .164 |
| Sexuality | Heterosexual  LGBTQ+ | 362  57 | 3.9382  3.7300 | 1.873 | 417 | .062 |
| Relationship | Single  Relationship | 224  211 | 3.9318  3.8830 | .645 | 433 | .520 |
| Ethnicity | White  Ethnic minority | 306  124 | 3.9010  3.9204 | -.231 | 428 | .818 |
| **Disability** | **No disability**  **Disability** | **375**  **58** | **3.9359**  **3.7132** | **2.009** | **431** | **.045*** |
| Caring responsibilities | Not a carer  Carer | 341  91 | 3.9334  3.8336 | 1.076 | 430 | .282 |
| PMQ | UK  Non-UK | 387  51 | 3.9095  3.9129 | -.028 | 436 | .977 |

* = statistically significant (p<.05)

Mean based on Likert scale: 1 = extremely unlikely, 2 = very unlikely, 3 = unlikely, 4 = neutral, 5 = likely, 6 = very likely, 7

= extremely likely

Appendix F. One-way ANOVA analyses comparing average likelihood of seeking any type of help across demographic groups: age group and career stage.

|  | **Between groups** | | | | |
| --- | --- | --- | --- | --- | --- |
| **Group** | **Sum of squares** | **df** | **Mean Square** | **F** | **p** |
| Age | 4.066 | 4 | 1.017 | 1.661 | .159 |
| **Career stage** | **6.897** | **5** | **1.379** | **2.272** | **.047*** |

* = statistically significant (p<.05)

When all sources of help were included, those without a disability were significantly more likely (mean 3.9) to seek help than those with a disability (mean 3.7), t (431) = 2.009, p=.045. Secondly, a one-way ANOVA revealed there was a significant difference between career stage groups and seeking any type of help, F (5, 434) = 2.272, p=.047. However, there were no significant results from Turkey’s HSD Test for multiple comparisons. No other results were significant.
